# Transactions of the American Dental Association

**Published:** 1890-04

**Authors:** 


### Transactions of The American Dental Association.

The minutes and proceedings of the twenty-ninth annual session of the American Dental Association, held in Saratoga Springs in August last, came to hand a short time ago. The volume is a very creditable one indeed; the matter is well prepared and arranged, as it always is from the faithful hands of the most excellent secretary aided by his co-laborers of the publication committee.

It makes a volume of one hundred and fifty-nine pages, and while not as large as the transactions of some former meetings of this body it can hardly be said to be of less importance.

The proceedings of our societies constitute an important department of our literature, and exhibit better and more fully than anything else the progress made in the profession from year to year; there the steps whether long or short are definitely seen. He who desires to keep up with the advance in dentistry can not afford to be without the transactions of the dental societies.

